# Effect of low temperature calcination on micro structure of hematite nanoparticles synthesized from waste iron source

**DOI:** 10.1016/j.heliyon.2024.e41030

**Published:** 2024-12-06

**Authors:** Juliya Khanam, Md Rashib Hasan, Bristy Biswas, Md Farid Ahmed, Sabrina Mostofa, Umme Sarmeen Akhtar, Md Kamal Hossain, Md Saiful Quddus, Samina Ahmed, Nahid Sharmin, Sharif Md Al-Reza

**Affiliations:** a Institute of Glass and Ceramic Research and Testing (IGCRT), Bangladesh Council of Scientific and Industrial Research (BCSIR), Dhaka, 1205, Bangladesh; b Department of Applied Chemistry and Chemical Engineering, Islamic University, Kushtia, Bangladesh; c BCSIR Dhaka Laboratories, Bangladesh Council of Scientific and Industrial Research (BCSIR), Dhaka, 1205, Bangladesh

**Keywords:** Hematite, Nanoparticle, Recycling, Structure, Morphology, Particle size

## Abstract

Hematite (α-Fe_2_O_3_) nanoparticles have been synthesized from waste source of iron which contains a prominent amount of iron (93.2 %) and investigated the effect of low temperature calcination. The two-step synthesis method involved preparing ferrous sulfate through acid leaching process followed by oxidation and calcination at temperatures ranging from 200 to 400 °C to produce the desired α-Fe_2_O_3_ in nano form. The structure, size and morphology of the hematite nanoparticles were characterized using various instrumental techniques including X-ray diffraction (XRD), X-ray photoelectron spectroscopy (XPS), Raman spectroscopy, Fourier transformed infrared (FTIR) spectroscopy, scanning electron microscopy (SEM), transmission electron microscopy (TEM), UV–visible diffuse reflectance spectroscopy (DRS), and a nanoparticle size analyzer. Hematite single phase was confirmed by XRD and the phase occurred at 200 °C might indicate the stability range of hematite under certain condition. The average crystal sizes were determined using Debye Scherer formula, modified Scherer formula, size strain plot equation and Halder-Wagner-Langford’s method and the results show that crystallite sizes decreased with increasing calcination temperature. XPS analysis confirmed the chemical state (Fe^3+^) and surface chemistry of the hematite nanoparticles calcined at 300 °C. Raman spectrum also supported that the nanoparticles were complete hematite phase and the intensity of all the features decreased with increasing calcination temperature which are consistence with the result obtained from XRD pattern. FTIR spectra of the samples also confirms the XRD results. Morphological analysis obtained from SEM and TEM images suggested the agglomerated irregular spherical nanoparticles with grain size 13.49 nm calcined at 300 °C. Band gap energy of the samples were calculated from DRS data and the values ranging from 2.30 to 2.42 eV which are slightly higher than the bulk (∼2.1eV). Particles size analysis have been carried out using DLS and Z-average particle size and poly dispersity index (PDI) were measured which indicate the particles are nearly same size (220–226 nm).

## 1 Introduction

Nanostructured materials research has been attracted much interest due to their wide range of important application and unique physico-chemical properties such as optical, electronic, magnetic properties that diverge greatly from their bulk materials as they have smaller grain size, quantum confinement effect, high sensitivity and high surface to volume ratio [[1], [2], [3], [4]]. Metal-oxide based nanoparticles have become a fascinating growing class of materials due to their exceptional stability, crystallinity, conductivity, biocompatibility and nontoxicity which open the doors for these types of materials to be potential candidates in several applications. Among the various metal oxide nanomaterial, iron oxide nanoparticles have attracted great attention due to their promising applications in gas sensors, magnetic resonance imaging, photo-electrochemical cells, solar cell, contrast agents, drug delivery, pigment, catalysis, environmental application and so on [1,[5], [6], [7], [8], [9]].

Iron oxides are particularly important because of their various applications and it exist in many form in nature including hematite (α-Fe_2_O_3_), maghemite (γ-Fe_2_O_3_) and magnetite (Fe_3_O_4_) in which hematite being probably most common phase. Hematite (α-Fe_2_O_3_) is a metal oxide n-type semiconductor with a band gap of ∼2.1eV which has a potential technological application such as catalysis, pigments, gas sensors, field effect transistor, photo electrolysis reactor, water treatment due to its non-toxicity, low processing cost, high resistance to corrosion, tunable biocompatibility and thermodynamic stability, environmental friendly [1,3,5,[10], [11], [12], [13], [14], [15], [16], [17]]. Various physical and chemical methods have been adopted for synthesis of hematite nanoparticles such as electron beam evaporation, sputtering technique, mechanical millings, high energy ball milling, co-precipitation, electrochemical, sol-gel method, ultrasonic irradiation, solvothermal method, combustion, solvent evaporation, spray pyrolysis, solvent extraction [[18], [19], [20]], microwave assisted synthesis [21], green synthesis, thermal transformation technique [22], polyol [23], calcination and so on [24,25]. Many researchers have used calcination and thermal decomposition methods for synthesis of hematite nanoparticles [12,26,27]. Xili et al. [27] synthesized hematite nanoparticles by hydrothermal-calcinaton route as calcination temperature ranging from 400 to 700 °C with tunable porous structure. J Sharmila Justus et al. [12] synthesized hematite nanoparticles in which the powders samples were subsequently calcined in air for 3h at three different temperatures ranging from 400 to 800 °C. Tati Nurhayati et al. [28] have obtained hematite nanoparticles using microwave assisted calcination method within the temperature range of 250–350 °C with FeCl_3_.6H_2_O and NaOH as precursors. They found from the XRD result that hematite formation began at 300 °C or slightly lower than 300 °C and the degree of crystallinity increases with increasing the calcination temperature. No phase formation occurred at calcination temperature 250 °C indicating the amorphous nature of the samples [28]. Here, we report the synthesis of hematite nanoparticles by oxidation-calcination of ferrous sulfate precursors obtained from waste condensed milk container through acid leaching process. Among the various chemical methods, low temperature calcination has several advantages for large scale synthesis of hematite nanoparticles such as easy and cost effective; no requirement for high pressure, energy, time, temperature and toxic chemicals as well as cheap available raw materials source. The starting source of raw material for the synthesis of hematite nanoparticles is waste condensed milk container having 93.2 % iron [8]. There are many source of iron containing material which used as a solid waste like mill scale, sludge, iron dust, iron scrape, condensed milk container, beverage can, refractories and sinter plant, steel industries and so on. Condensed milk container can be used in many tea stall, restaurants and household purposes in Bangladesh. After using the inner material, the remaining containers are just thrown out drastically in the environment. Solid waste dumping in open spaces is a great concern for environmental issues as the environment and how to save it has been an area of prime focus for the entire world community [29]. So, the implementation of appropriate solid waste management should be taken into account in order to find solutions that are economically appealing and environmentally sustainable [30,31]. Recycling of solid waste products as well as household wastes have been converted into developed and advanced products by attentive manipulation of synthetic condition. The continued development of recycling and recovery technologies for solid waste materials are considered as economically and environmentally viable when the maximum utilization of the waste products have done into value added products by appropriate method.

Based on earlier studies, many researchers have reported that hematite typically forms around 300 °C. However, the recent findings indicating that hematite can form at 200 °C via the Combustion-Calcination method which open up new avenues for research [32]. The research report here in focusing on the synthesizing hematite nanoparticles from waste condensed milk container through a simple acid leaching method with low temperature calcination which is environmentally friendly. This approach not only utilizes waste materials but also potentially reduces energy consumption compared to traditional high-temperature synthesis methods. The other purpose of this research is to create opportunity for the small entrepreneur so that they can develop an industry in Bangladesh to achieve the vision and mission for sustainable development. The investigation of structural, morphological, surface chemistry and optical property have been carried out using different technique such as XRD, FTIR, RAMAN, SEM, TEM, DLS, DRS and XPS.

## 2 Methodology

### 2.1 Materials and reagents

Locally available waste condensed milk containers were used as a primary source of iron which contain 93.2 % iron [8]. Sulphuric acid, H₂SO₄ (Merck, Germany) and Sodium nitrate, NaNO₃ (Merck Germany) were used as received without further purification for the synthesis of hematite (α-Fe_2_O_3_) nanoparticles.

### 2.2 Synthesis of hematite

Hematite (α-Fe_2_O_3_) nanoparticles were synthesized from ferrous sulfate by oxidation-calcination method in which waste condensed milk container cans have been used as a source of iron content for the preparation of ferrous sulfate through acid leaching process [8]. The collected cans were first washed properly and then dried and cut into small pieces for the maximum acid leaching followed by crystallization. A mixture of specified amount of dried ferrous sulfate, FeSO_4_ and sodium nitrate, NaNO_3_ were taken in porcelain crucible, according to a designed weight ratio of FeSO₄:NaNO₃ = 1:0.02 was maintained. Then the mixture was heated slowly in a burner for a certain period of time and again reheated vigorously for 30 min. The resultant burned product of FeSO₄ was cooled and grinded and finally the mixture was calcined at 200, 300 and 400 °C for 4 h in a muffle furnace. The product obtained from calcination was cooled, grounded and washed several times to reduce the impurities and soluble component. After that, the washed samples were filtered and dried in an oven at 110 °C for 2 h. To get the small grain size, the obtained hematite was then again grounded properly.

### 2.3 Characterization techniques

The structural analysis and phase identification of the samples were carried out by X-ray diffraction (XRD) technique using SmartLab SE, Rigaku, Japan with CuKα radiation (λ = 1.540898 Å) source, and 2 theta range of 10°–80°. Fourier Transform Infrared (FTIR) spectra has been recorded in the range of 400-4000 cm^−1^ by using NICOLET iS5, Thermofisher. Surface morphology, grain size and the elemental analysis of the hematite nanoparticles were investigated by scanning electron microscopy (MA15 VP-SEM, Carl Zeiss Evo, UK) and transmission electron microscopy (JEM-2100 Plus, Japan) with energy dispersive spectroscopy (EDS). Raman spectra were collected by a macroRAM, HORIBA Scientific, Japan Raman microscope with 633 nm excitation light in the range 70–1000 cm^−1^. UV–Vis diffuse reflectance spectra (DRS) were obtained by UV–Vis spectrometer PerkinElmer, Lamda 1050+ USA, equipped with an integrating sphere and the baseline correction was performed using a calibrated reference sample of powdered barium sulfate (BaSO_4_). The spectra were recorded at room temperature in the wavelength range of 200–800 nm. Surface chemistry and the chemical state of the synthesized nano particles were explored by X-ray photoelectron spectroscopy (XPS), K-alpha, Thermo Scientific, by using a monochromatic source of Al K-alpha (λ = 1486.69 eV) X-ray source. The calibration of the binding energy curves were performed using C 1s peak at 284.8 eV.

## 3 Results and discussion

### 3.1 XRD analysis

**Fig. 1:**
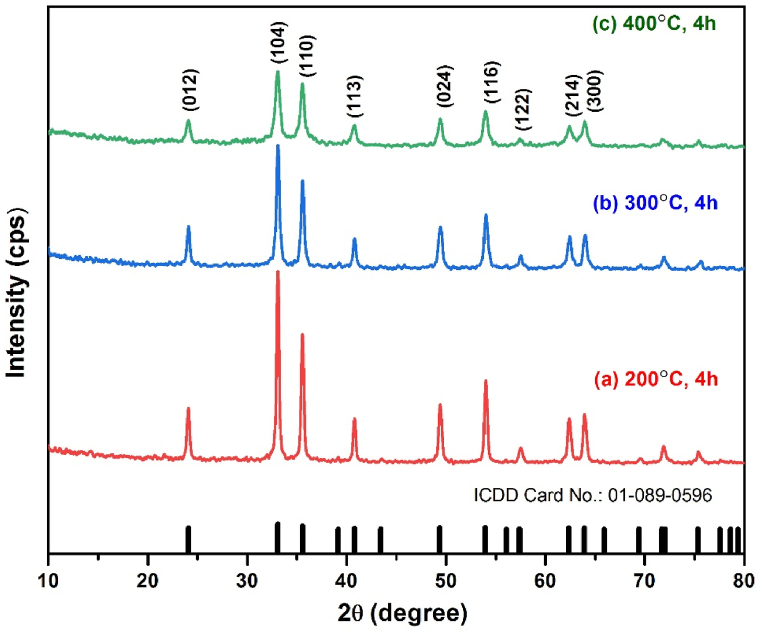
XRD pattern of α-Fe_2_O_3_ nanoparticles at different calcination temperatures.

**Fig. 2:**
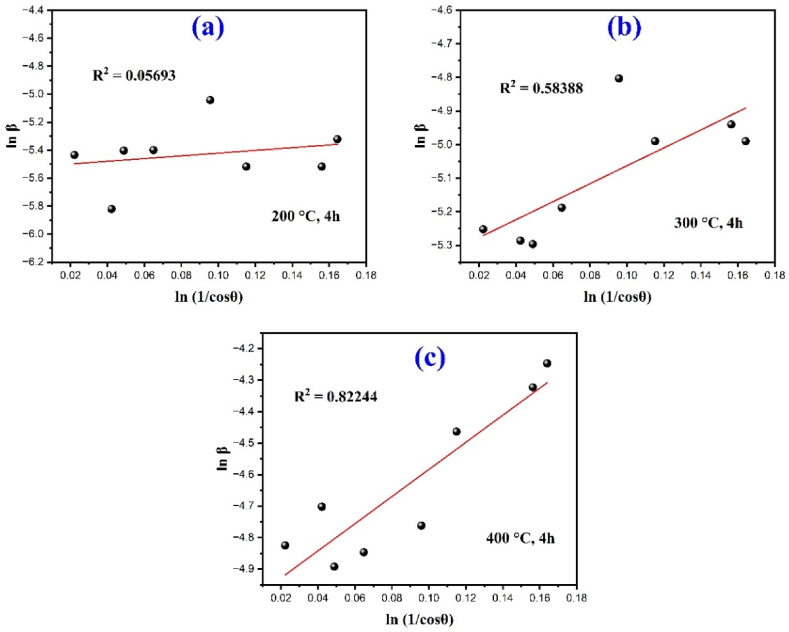
The least square modified Scherer equation plot of XRD.

The crystal structure and the purity of the synthesized iron oxide nanoparticles calcined at low temperature ranging from 200, 300 and 400 °C were investigated by XRD as represented in Fig. 1. All the diffraction peaks of the calcined samples were found to be well matched with the standard α-Fe_2_O_3_ reflections (JCPDS card No. 01-089-0596) representing that the α-Fe_2_O_3_ nanoparticles are highly crystalline structure. No other diffraction line corresponding to other phases has been observed, indicating high purity of the samples. It has been also showed that there have a significant change in peaks variation due to different calcination temperature. It is clearly noticed that as the calcination temperature increases, the width of the peaks have been broadened indicating the small crystallite size. Crystallite size increases or decreases is depend on according to ordering inside the material (density of the localized states). Structural and textural properties are normally changed with increase of calcination temperature [33]. The crystallinity of the samples is directly related to the crystallite size [34]. For the identification of level of crystallinity, the average crystal size have been determined by various method including classical Debye-Scherer Formula (CS), the least square modified Scherer Formula (MS), Size strain plot (SSP) method, Williamson Hall (WH) method and Halder Wagner Langford’s method (HWL). Moreover, the lattice parameters (a, b, and c) have been calculated by the following expression, where d is the inter planar distance, a and c are the lattice parameters and h, k, l are the Miller indices [35]. The results are shown in Table 1 (Fig. 2).

(1) $$d_{hkl}=\frac{1}{\sqrt{\frac{4}{3a^{2\;}}\left(h^{2}+k^{2}+l^{2}\right)\;\pm \;\frac{l^{2}}{c^{2}}}}$$

**Table 1:** Determination of lattice parameter and crystal size by different method.

Sample	Crystal Size (nm)				Lattice parameter	
	D_CS_	D_MS_	D_SSP_	D_HWL_	a (Å) = b (Å)	c (Å)
200 °C, 4h	29.73	34.57	31.87	31.73	5.04235	13.79344
300 °C. 4h	21.14	28.63	29.01	28.65	5.03890	13.78402
400 °C, 4h	13.07	20.88	19.28	18.76	5.03799	13.78402

#### 3.1.1 Determination of crystal size

##### 3.1.1.1 Classical Scherer (CS) and modified Scherer Formula (MS)

XRD can be utilized to evaluate peak broadening in the nano crystals with crystallite size and intrinsic lattice strain effects due to dislocation. The peak broadening basically consists of two parts, physical broadening and instrumental broadening which have been measured as full width at half maxima (FWHM). The crystallite size of the α-Fe_2_O_3_ nanoparticles was measured by the X-ray broadening method using Debye-Scherer Eq. (2) [36,37].

(2) $$D_{CS}=\frac{k\lambda }{\beta \;\cos\;\theta }$$

Where *K* refers to the shape factor (0.9), λ is the wavelength of the incident CuKα = 1.5406 Å radiation, β is the full width at half maxima (FWHM) and θ is the Bragg diffraction angle. By rearranging Eq. (2) integral breadth can be obtained.

(3) $$\beta =\frac{k\lambda }{D_{CS}\;\cos\;\theta }$$

Modified Scherer equation also obtained by rearranging Eq. (3) and taking logarithm on both side as follows:

(4) $$\ln\;\beta =\ln\left(k\lambda /D_{M-S}\right)+\ln\left(1/\cos\;\theta \right)$$

By linear plot of ln(1/cosθ) vs. lnβ , the slope and intercept of the fitted line will obtain as modified Scherer equation. Using the obtained intercept, the average crystal size can be calculated by Eq. (4). by following relation:

$$D_{MS\;}=k\lambda /e^{intercept}$$

##### 3.1.1.2 Size-strain plot (SSP)

Size strain plot is one of the peak profile analysis method which considers that XRD peak profile is a combination of Lorentzian function and Gaussian function, where size broadened profile is labeled as Lorentz function and strain broadened profile is labeled as Gaussian function and it expressed as follow [38,39]:

(5) (dhkl.βhkl.cosθ)2=kλ/DSSP(dhkl2.βhkl.cosθ)+ℇ24

**Fig. 3:**
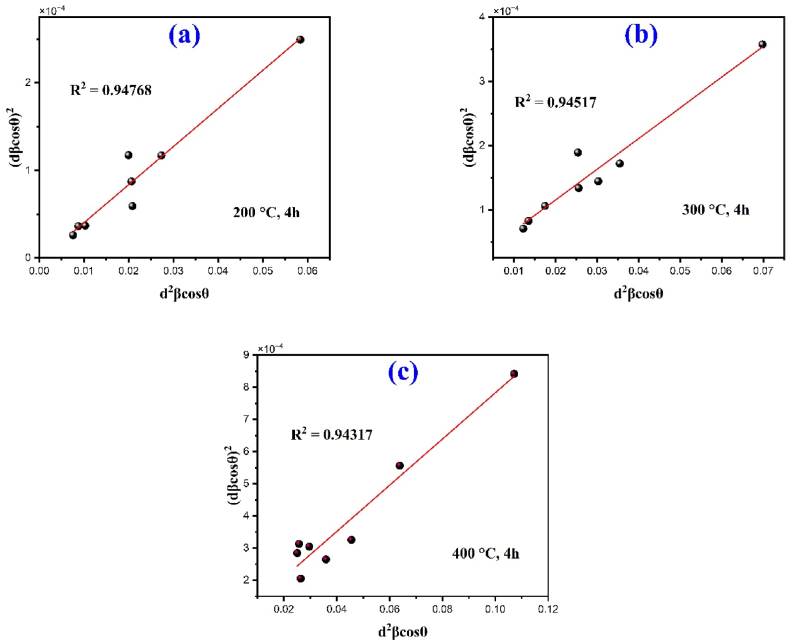
Size strain plot of the nanoparticles.

Where d_hkl_ is the latticle distance between the (hkl) planes. By using the above Eq. (5), a plot is drawn with $(d_{hkl}^{2}{.\beta }_{hkl}.\cos\;\theta$) vs. $\left(d_{hkl}{.\beta }_{hkl}.\cos\;\theta \right)$^2^ corresponding to each diffraction peak, which is shown in Fig. 3. The average crystallite size D, will be obtained from the linear fit slope as given below:

(6) $$D_{SSP=}\frac{k\lambda }{slope}$$

##### 3.1.1.3 Halder – Wagner Langford’s (HWL) method

The average crystallite size was also determined using the HWL method from the integral breadths, β of the XRD data. In the SSP method the size broadening and the strain broadening of the XRD peak profile have been assumed as a function of Lorentzian and Gaussian function respectively but actually XRD peak is neither Lorentzian function nor Gaussian function. Since the Gaussian function matches the XRD peak region well, but without a match, its tail falls down very rapidly and on the other hand, tails of the profile fits quite well with Lorentz function, but that fails to match the XRD peak region. Therefore, to overcome this Halder-Wagner Langford method is applied in which the peak broadening is a symmetric Voigt function and convolution of Gaussian and Lorentz functions are used. According to Halder-Wagner method the relation between the crystallite size and the lattice strain is expressed as [40,41]:

(7) $${\left(\frac{\beta^{*}}{d^{*}}\right)}^{2}=\frac{K}{D}\left(\frac{\beta^{*}}{d^{{*}^{2}}}\right)+{2ℇ}^{2}$$

Here β∗ = βcosθ/λ and d∗ = 2sinθ/λ, λ is the X-ray wavelength. By putting the value of β∗ and d∗ in Eq. (7) and it can be modified as:

(8) $${\left(\frac{\beta \;\cos\;\theta }{\sin\;\theta }\right)}^{2}=\frac{k\lambda }{D_{HWL}}\frac{\beta \;\cos\;\theta }{\sin^{2}\;\theta }+16{ℇ}^{2}$$

Eq. (8) can also be written as:

(9) $${\left(\frac{\beta }{\tan\;\theta }\right)}^{2}=\frac{k\lambda }{D_{HWL}}\frac{\beta \;\cos\;\theta }{\sin^{2}\;\theta }+16{ℇ}^{2}$$

**Fig. 4:**
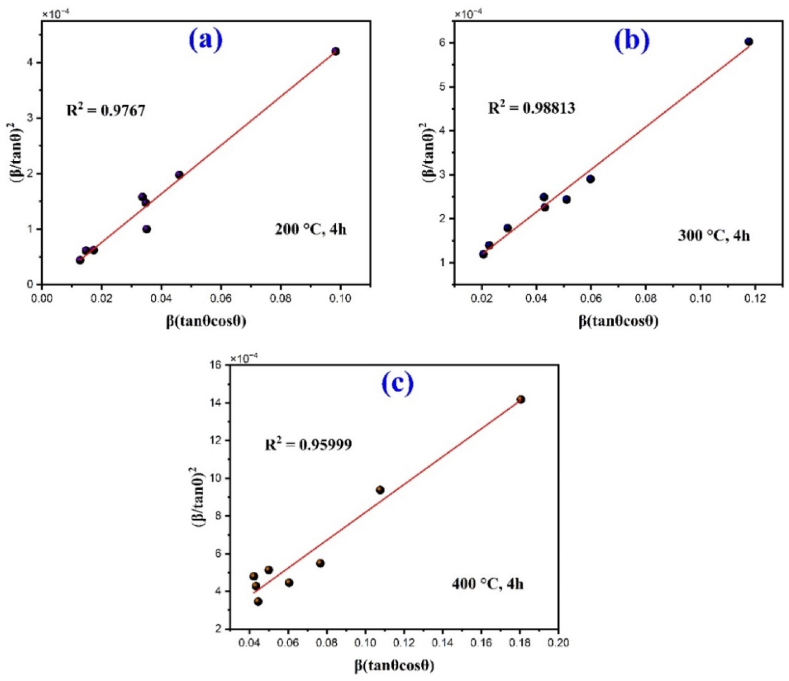
Halder-Wagner plot of the α-Fe_2_O_3_ nanoparticles.

Now plot the graph $\frac{\beta \;\cos\;\theta }{\sin^{2}\;\theta }$ vs. $\left(\frac{\beta }{\tan\;\theta }\right)$^2^ along Y-axis for all reflection data, it shows a linear fit with straight line equation where the slope of the line provides the average crystal size $D_{HWL}$ and the intercept gives the strain ℇ as shown in Fig. 4.

The calculated average crystal size determined by different method as shown in Table 1 that the crystallite size decreases as the calcination temperature increases and the calculated values have been changed in different method. Crystallite size ranges vary from 13.07 nm to 29.73 nm for classical Scherrer formula (CS), 20.88 nm–34.52 nm for modified Scherer formula (MS), 19.28 nm–31.87 nm for the size strain plot method (SSP) and 18.76 nm–31.87 nm for Halder Wagner Langfords model (HWL) for calcination temperature of 400, 300 and 200 °C. The applied three methods including MS, SSP and HWL give nearly same crystallite size as compared to another method CS in which crystallite size slightly smaller than the other methods. Conversely, the HWL model exhibits the higher degree of accuracy with fitted data as compared to MS method and SSP model and the $R^{2}$ values are 0.9767, 0.9881 and 0.9599 for 200, 300 and 400 °C respectively.

### 3.2 SEM and EDS analysis

**Fig. 5:**
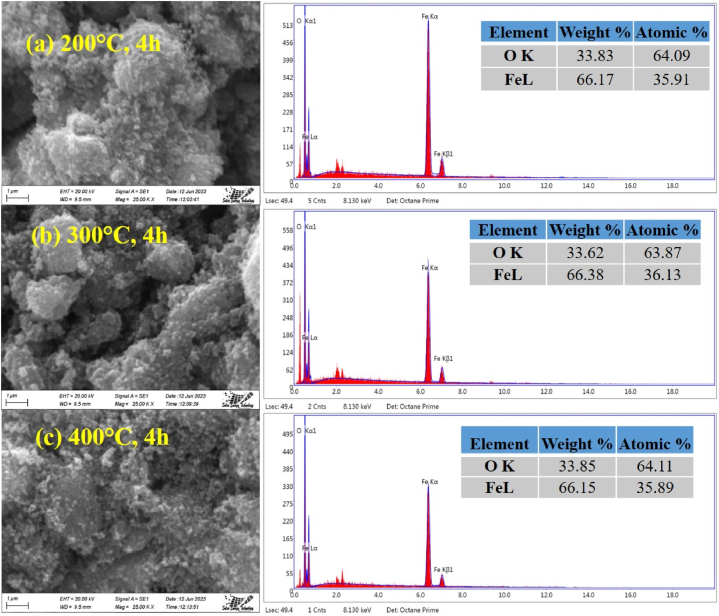
SEM images (left) and EDS (right) data of α-Fe_2_O_3_ nanoparticles calcined at different temperature.

The characterization of hematite nanoparticles calcined at different temperatures were also carried out using SEM and EDS analysis as depicted in Fig. 5. It has been found that the particles are seriously agglomerated and polygonal in shape [42]. Nevertheless, the precise shape and size of the individual nanoparticles could not be decided accurately owing to the formation of large aggregate or cluster [43] as shown in Fig. 5. The image of the synthesis materials revealed an aggregated nature with hematite particles predominating among the various crystal morphologies. EDS spectra also showed the purity of the nanoparticles by getting the three peaks of iron (Fe) and one peak of oxygen (O) for all the synthesized of α-Fe_2_O_3_ nanoparticles [24].

### 3.3 FTIR spectroscopic analysis

**Fig. 6:**
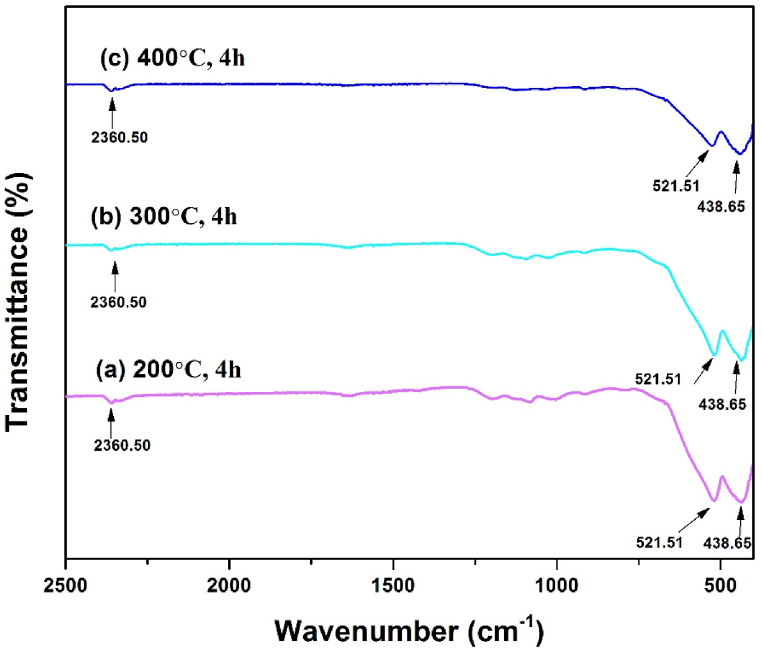
FTIR spectra of α-Fe_2_O_3_ nanoparticles at various temperature.

The functional group and the metal-oxygen bond formation of the hematite nanoparticles has been investigated by Fourier Transform Infrared spectrometer. Fig. 6 represents the absorption data of low temperature calcined hematite nanoparticles which confirmed that the synthesized samples are IR active. The prominent absorption bands observed at 438.65 and 521.51 cm^−1^ are due to metal-oxygen stretching vibration [1,2,42,44,45]. The weak absorption peak observed at 2360 cm^−1^ is an asymmetric stretching peak of CO_2_ in the air [46].

### 3.4 Raman spectroscopic analysis

**Fig. 7:**
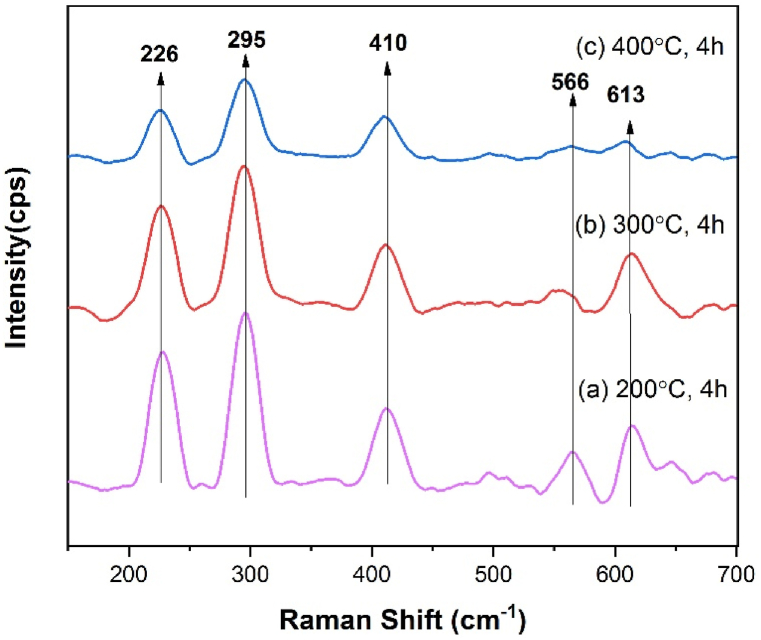
Raman Spectra of the hematite nanoparticles calcined at different temperature.

Raman spectra of the hematite nanoparticles are shown in Fig. 7. Seven phonon lines are expected in hematite single crystal structure in which two modes of A1g at about 226 and 496 cm^−1^ and five modes of Eg at about 245, 293, 298, 410, 613 cm^−1^ [35,42,47,48]. The significant four vibration peaks centered at around 226 (A1g), 295 (Eg), 410 (Eg) and 613 (Eg) cm^−1^ are different mode of vibration [49]. A broad band at around 566 cm^−1^ for the sample calcined at 200 °C, 4 h is due to the amorphous iron oxides [50]. It was clearly observed from Fig. 7 that the intensity of the all the Raman peaks decreased with increasing calcination temperature which also indicated the lower crystallite size confirmed by XRD analysis.

### 3.5 TEM and EDS analysis

**Fig. 8:**
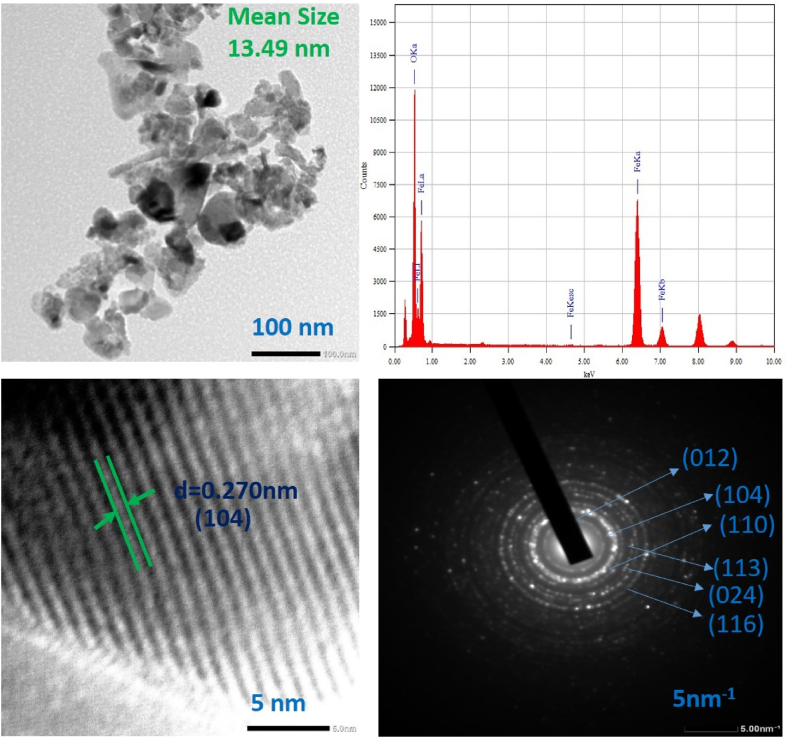
TEM image with EDS data and selected area diffraction pattern of α-Fe_2_O_3_ nanoparticles calcined at 300 °C, 4 h.

The TEM image with EDS analysis of α-Fe_2_O_3_ nanoparticles calcined at 300 °C, 4 h are depicted in Fig. 8. The surface morphology of the nanoparticles are mostly agglomerated in nature which are the common tendency of nanoparticles. The average particle size is 13.49 nm. The purity of the nanomaterials were also examined by EDS analysis with the existence of elemental confirmation which indicate that synthesized hematite nano materials are composed of Fe and O. This data is in good agreement with the data obtained from SEM-EDS. The high resolution TEM (HRTEM) gives the crystal lattice fringe spacing between the crystal lattice plane (104) and the calculated value is 0.270 nm. HRTEM also shows uniform lattice crystal structure without detectable defects [45]. The selected area electron diffraction (SAED) pattern also shows the poly crystalline behavior of the hematite nanoparticles with cubic crystal structure.

### 3.6 Optical properties

**Fig. 9:**
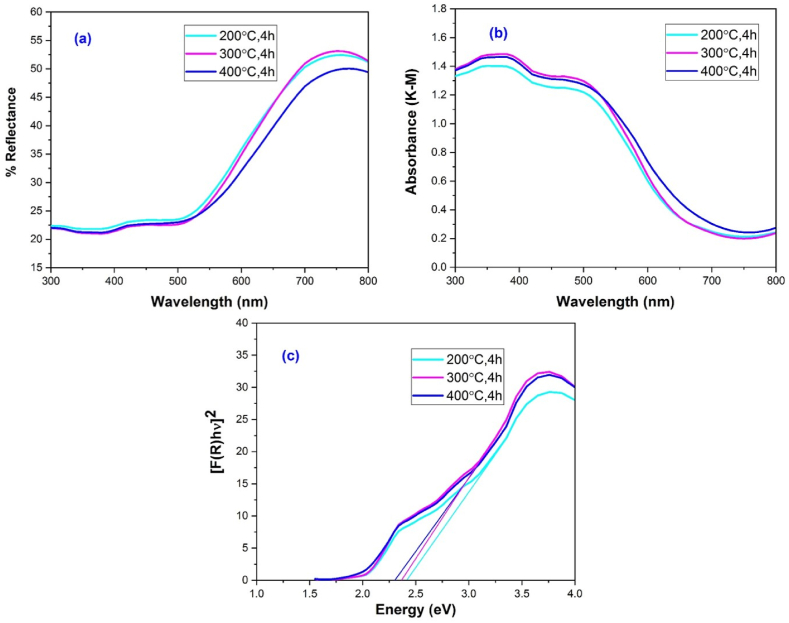
The optical properties of hematite nanoparticles calcined at different temperature (a) UV–Vis diffuse reflectance spectra (b) UV–Vis absorbance spectra (c) Band gap energy.

The optical properties of the synthesized hematite nanoparticles measured at the wavelength range of 200–800 nm have been shown in Fig. 9. By using Kubelka-Munk function, absorption data obtained from the reflectance was used to evaluate the optical nature of synthesized hematite nanoparticles. The spectrum shows four absorption band in the UV–visible region as shown in Fig. 9(b). The first absorbed region was found to be in the range of 300–400 nm and it has been centered at 347 nm which is basically due to the metal charge transfer transition between valence band and conduction band. The second absorption band was located at the wavelength region of 401.57 nm is attributed at ligand to field transition due to the contribution of Fe^3+^ ligand field transition. The maximum absorption band was recorded in the visible light region at 518 nm could be assigned as pair excitation between neighboring Fe^3+^ - Fe^3+^ cation [[5], [6], [7],^51^]. The other absorption band observed at 662.25 nm is responsible for ligand field transition. The optical band gap energy (E_g_) of the synthesized α-Fe_2_O_3_ nanoparticles was estimated by using Kubelka-Munk function.

(10) $$F\;\left(R\right)=\frac{{\left(1-R\right)}^{2}}{2R}=\frac{K}{S}$$

Where R is the absolute reflectance of the sample, K is the molar absorption coefficient and S is the scattering coefficient. The band energy gap was plotted as (F(R) hv)^2^ vs hv which is shown in Fig. 9(c). The increase of calcination temperature having low crystallite size and grain size have affected the low band gap energy value within the range of 2.30–2.42 eV as recorded in Table 2. Microstructural characteristic is greatly affected by the E_g_ value and it is influenced by various factors such as surface/interface structure, grain size, crystal quality, lattice parameters, lattice strain, carrier concentrations, defect structure, and chemical composition [52]. Eg slighty shifts to 2.30–2.42 eV compared to that of pure α-Fe_2_O_3_ (2.1 eV) with decreasing calcination temperature. The decreasing is attributed to the size effect, as the crystallite size of the samples decreased with increasing the calcination temperature [26]. XRD measurements clearly indicate that single phase formation is commenced for 200 °C with Eg value 2.42 and increasing calcination temperatures have significant effect on Eg values.

**Table 2:** Optical band gap energy of α-Fe_2_O_3_ nano particles calcined at different temperature.

Sample	Band gap, E_g_ (eV)
200 °C, 4h	2.42
300 °C, 4h	2.37
400 °C, 4h	2.30

### 3.7 Particle size analysis by DLS

**Fig. 10:**
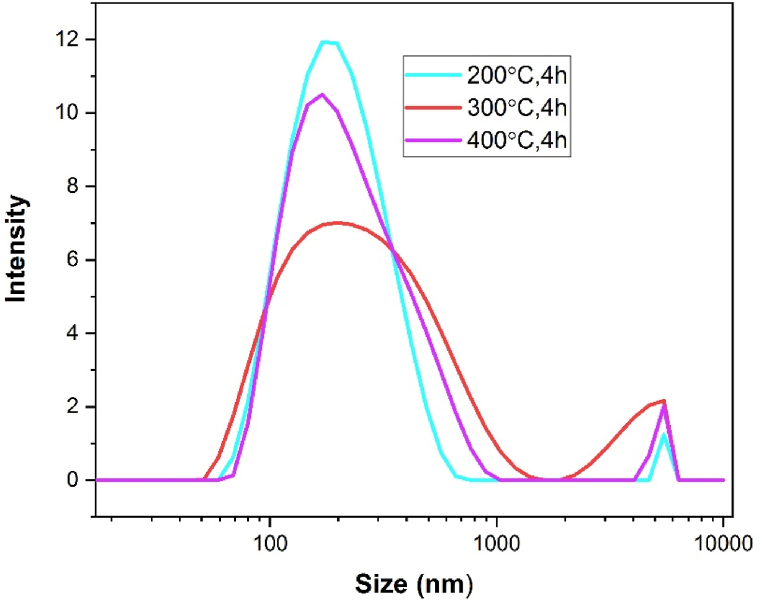
Particle size distribution of α-Fe_2_O_3_ nanoparticles calcined at different temperature.

Particle size distribution of hematite nanoparticles measured by dynamic light scattering method (DLS) are shown in Fig. 10. The hydrodynamic diameter ${\bar{D}}_{h}$ was calculated from the Z-average translation diffusion coefficient $\bar{D}$ through the Stokes-Einstein Eq [53].:

(11) $${\bar{D}}_{h}=\frac{k_{B}T}{3\pi \eta \bar{D}}$$

Where $k_{B}$ is the Boltzmann constant and η is the solvent viscosity at temperature T. The Z-average apparent hydrodynamic diameter ${\bar{D}}_{h}$ and polydispersity index (PDI) are recorded in Table 3. It is clearly seen from the table that the Z-average and the PDI values of all the hematite nanoparticles are nearly same which also indicates the poly dispersity of the particles. The mean particle size determined by dynamic light scattering (DLS) is larger than the crystallite size calculated from X-ray diffraction (XRD) data. This discrepancy arises because DLS measures the hydrodynamic diameter, reflecting the time-dependent fluctuations in scattered light intensity caused by the Brownian motion of the particles. Consequently, higher hydrodynamic particle size values suggest that the nanoparticles tend to agglomerate, forming larger clusters when dispersed in a solvent [33]. Agglomeration or aggregation often renders nanoparticles less stable, resulting in a more heterogeneous mixture that can lead to inconsistent properties and performance. In contrast, homogeneous particles tend to exhibit greater stability [54]. The PDI values and the average particles size of the hematite nanoparticles indicate that the synthesized samples are notably polydisperse. There is no significant change in particle sizes with increasing temperature.

**Table 3:** Z-average hydrodynamic diameter ${\bar{D}}_{h}$ and polydispersity index (PDI) of α-Fe_2_O_3_ nanoparticles calcined at low temperature and time.

Sample	${\bar{D}}_{h}$ nm	PDI
200 °C, 4h	220	0.3532
300 °C, 4h	226	0.3953
400 °C, 4h	224	0.3534

### 3.8 Chemical state analysis by XPS

**Fig. 11:**
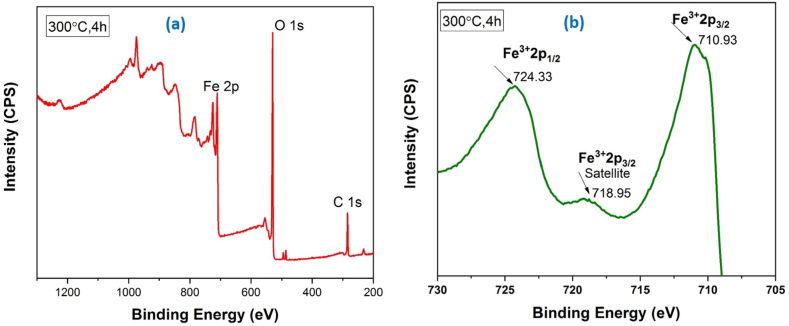
XPS data of α-Fe_2_O_3_ nanoparticles (a) survey spectrum data calcined at 300 °C, 4 h (b) Fe 2p spectrum calcined at 300 °C, 4 h.

X-ray photoelectron spectroscopy (XPS) is the suitable technique for identifying precisely the oxidation state of Fe and O atoms of hematite nanoparticles. The survey spectrum and the high resolution XPS spectra of Fe 2p calcined at 300 °C, 4h are shown in Fig. 11. The nanoparticles were primarily composed of Fe and O atoms. The peak position corresponding to Fe 2p has been searched in the binding energy range 705–730. The peaks position having binding energy 710.93 eV and 724.33 eV representing the Fe^3+^2p 3/2 and Fe^3+^2p 1/2 with a satellite peak observed at 718.95 eV also indicate the Fe^3+^2p 3/2 agreeing very closely the previously reported [8,44,[55], [56], [57], [58]]. Hematite and maghemite exhibit nearly identical spectra. However, the difference in lattice structure (cubic vs.hexagonal) does not impact on chemical shift. In survey spectrum C 1s peak observed at 284.8 eV is responsible for adventitious carbon contamination. Therefore, the XPS studies indicate iron in Fe^3+^ which confirm the formation of Fe_2_O_3_ as well as the crystal phase α-Fe_2_O_3_ has already identified by XRD.

## 4 Comparative study

A detailed comparison of hematite nanoparticles synthesized by different methods is presented in Table 4, highlighting the variations in synthesis conditions.

**Table 4:** Comparison of current research with the literature.

Sample	Synthesis procedure	Temperature (°C)	Time (h)	Ref.
α-Fe_2_O_3_	Green and Chemical method	200, 300 and 500	2,4 and 5	[59]
α-Fe_2_O_3_	Hydrothermal method	160 and 180	10 and 12	[60]
α-Fe_2_O_3_	Green synthesis	60	–	[61]
α-Fe_2_O_3_/Fe_3_O_4_	Combustion-Calcination	200, 250,300, 350 and 400	2	[32]
α-Fe_2_O_3_	Calcination	800 and 900	1	[62]
α-Fe_2_O_3_	Oxidation-Calcination	200, 300 and 400	4	Present work

## 5 Conclusion

Hematite (α-Fe_2_O_3_) nanoparticles have been successfully synthesized from waste condensed milk containers and the effect of low temperature calcination on the synthesized samples have also been studied. Structural, morphological and optical nature of the α-Fe_2_O_3_ nanoparticles were investigated by different characterization techniques. Hematite phase can be successfully prepared at 200 °C, as confirmed by various structural analyses. Crystallite size measurements indicate that, with increasing calcination temperature, the crystallite size decreases, ranging from 13.07 to 34.57 nm across different methods. The morphological analysis and elemental composition of the synthesized α-Fe₂O₃ nanoparticles reveal noticeable agglomeration behavior, confirming that the nanoparticles are composed solely of iron (Fe) and oxygen (O). The XPS data also provides the surface chemistry and oxidation state of Fe 2p and O1s which also supported the purity of the hematite nanoparticles calcined at 300 °C, 4h. Optical properties and band gap analysis measured from Kubelka-Munk function exhibit that the optical band gap energy slightly decreases with increasing temperature which can be used as a photo catalytic dye degradation. The mean particle size are quite similar for distinct calcination temperature 200, 300 and 400 °C with the values of 220, 226 and 224 nm respectively. In conclusion, the hematite (α-Fe_2_O_3_) nanoparticles synthesized from waste condensed milk container show great potential as a versatile material for various application such as photo catalyst, pigment, sensors etc.

## CRediT authorship contribution statement

**Juliya Khanam:** Writing – original draft, Methodology, Investigation, Formal analysis, Conceptualization. **Md Rashib Hasan:** Methodology. **Bristy Biswas:** Formal analysis. **Md Farid Ahmed:** Formal analysis. **Sabrina Mostofa:** Formal analysis. **Umme Sarmeen Akhtar:** Formal analysis. **Md Kamal Hossain:** Formal analysis. **Md Saiful Quddus:** Formal analysis. **Samina Ahmed:** Visualization, Supervision. **Nahid Sharmin:** Writing – review & editing, Supervision. **Sharif Md Al-Reza:** Supervision.

## Data availability statement

Data will be made available on request from authors.

## Declaration of generative AI

No AI software was used to prepare the manuscript.

## Declaration of competing interest

The authors declare that they have no known competing financial interests or personal relationships that could have appeared to influence the work reported in this paper.
